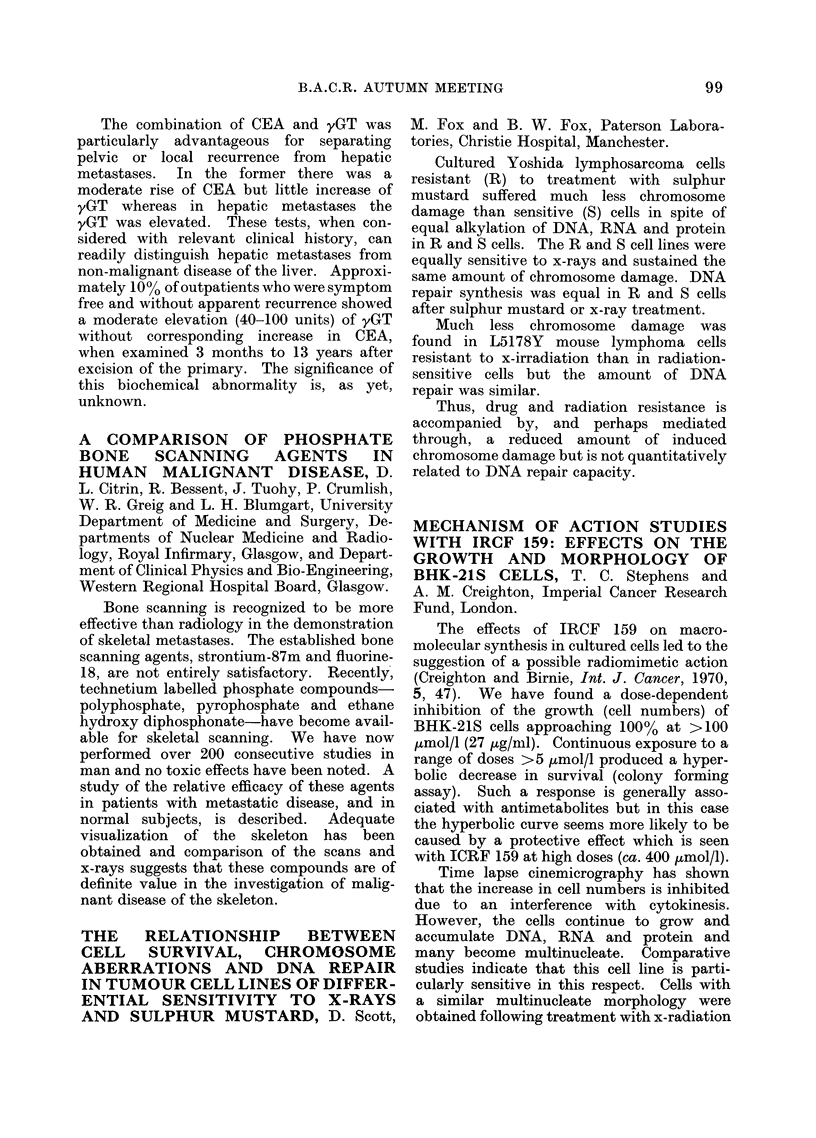# Proceedings: The relationship between cell survival, chromosome aberrations and DNA repair in tumour cell lines of differential sensitivity to X-rays and sulphur mustard.

**DOI:** 10.1038/bjc.1974.38

**Published:** 1974-01

**Authors:** D. Scott, M. Fox, B. W. Fox


**THE RELATIONSHIP BETWEEN CELL SURVIVAL, CHROMOSOME ABERRATIONS AND DNA REPAIR IN TUMOUR CELL LINES OF DIFFERENTIAL SENSITIVITY TO X-RAYS AND SULPHUR MUSTARD,** D. Scott, M. Fox and B. W. Fox, Paterson Laboratories, Christie Hospital, Manchester.

Cultured Yoshida lymphosarcoma cells resistant (R) to treatment with sulphur mustard suffered much less chromosome damage than sensitive (S) cells in spite of equal alkylation of DNA, RNA and protein in R and S cells. The R and S cell lines were equally sensitive to x-rays and sustained the same amount of chromosome damage. DNA repair synthesis was equal in R and S cells after sulphur mustard or x-ray treatment.

Much less chromosome damage was found in L5178Y mouse lymphoma cells resistant to x-irradiation than in radiation-sensitive cells but the amount of DNA repair was similar.

Thus, drug and radiation resistance is accompanied by, and perhaps mediated through, a reduced amount of induced chromosome damage but is not quantitatively related to DNA repair capacity.